# Association between Immunophenotypic Parameters and Molecular Alterations in Acute Myeloid Leukemia

**DOI:** 10.3390/biomedicines11041098

**Published:** 2023-04-05

**Authors:** Flávia Melo Cunha de Pinho Pessoa, Caio Bezerra Machado, Igor Valentim Barreto, Giulia Freire Sampaio, Deivide de Sousa Oliveira, Rodrigo Monteiro Ribeiro, Germison Silva Lopes, Maria Elisabete Amaral de Moraes, Manoel Odorico de Moraes Filho, Lucas Eduardo Botelho de Souza, André Salim Khayat, Caroline Aquino Moreira-Nunes

**Affiliations:** 1 Department of Medicine, Pharmacogenetics Laboratory, Drug Research and Development Center (NPDM), Federal University of Ceará, Fortaleza 60430-275, CE, Brazil; 2 Unichristus University Center, Faculty of Biomedicine, Fortaleza 60430-275, CE, Brazil; 3 Department of Hematology, Fortaleza General Hospital (HGF), Fortaleza 60150-160, CE, Brazil; 4 Department of Hematology, César Cals General Hospital, Fortaleza 60015-152, CE, Brazil; 5 Center for Cell-Based Therapy, Regional Blood Center of Ribeirão Preto, University of São Paulo, São Paulo 14040-900, SP, Brazil; 6 Department of Biological Sciences, Oncology Research Center, Federal University of Pará, Belém 66073-005, PA, Brazil

**Keywords:** acute myeloid leukemia, genetic alterations, immunophenotyping, prognosis

## Abstract

Acute myeloid leukemia (AML) is a hematologic malignancy that occurs due to alterations such as genetic mutations, chromosomal translocations, or changes in molecular levels. These alterations can accumulate in stem cells and hematopoietic progenitors, leading to the development of AML, which has a prevalence of 80% of acute leukemias in the adult population. Recurrent cytogenetic abnormalities, in addition to mediating leukemogenesis onset, participate in its evolution and can be used as established diagnostic and prognostic markers. Most of these mutations confer resistance to the traditionally used treatments and, therefore, the aberrant protein products are also considered therapeutic targets. The surface antigens of a cell are characterized through immunophenotyping, which has the ability to identify and differentiate the degrees of maturation and the lineage of the target cell, whether benign or malignant. With this, we seek to establish a relationship according to the molecular aberrations and immunophenotypic alterations that cells with AML present.

## 1. Introduction

Acute myeloid leukemia (AML) is a malignant disorder of hematopoietic stem cells that emerges due to genetic alterations, such as genetic mutations, chromosomal translocations, or changes in molecular levels. This disease is characterized by clonal expansion of abnormally differentiated blasts of a myeloid lineage [1,2,3]. The increased proliferation of immature myeloid cells causes the accumulation of blasts and impairs normal hematopoiesis, which may lead to hemorrhages, anemia, and severe and persistent infections. These are known to be the most common symptoms in the majority of patients with acute leukemias [3,4].

Although the exact cause of genetic mutations is unclear, the development of AML is associated with some risk factors, such as exposure to radiation, pesticides, chemotherapeutic agents, and smoking. AML can also evolve from other hematological diseases, including myelodysplastic syndrome (MDS), myeloproliferative syndromes (MPS), and aplastic anemia. AML risk also increases for people with some congenital disorders, such as Down syndrome and Bloom syndrome [3,5,6,7].

AML is the most frequent leukemia in adults, accounting for about 80% of acute leukemia cases in this population. It mostly affects male patients, with incidence rates increasing with age. Per year, the number of new AML cases is 4.2 per 100,000 population [3,4,8,9,10].

## 2. Genetic Alterations in AML

In AML, recurrent cytogenetic abnormalities are established diagnostic and prognostic markers; they can be therapeutically targetable since most of these mutations confer resistance to the therapeutic options used traditionally [11,12,13].

**Figure 1:**
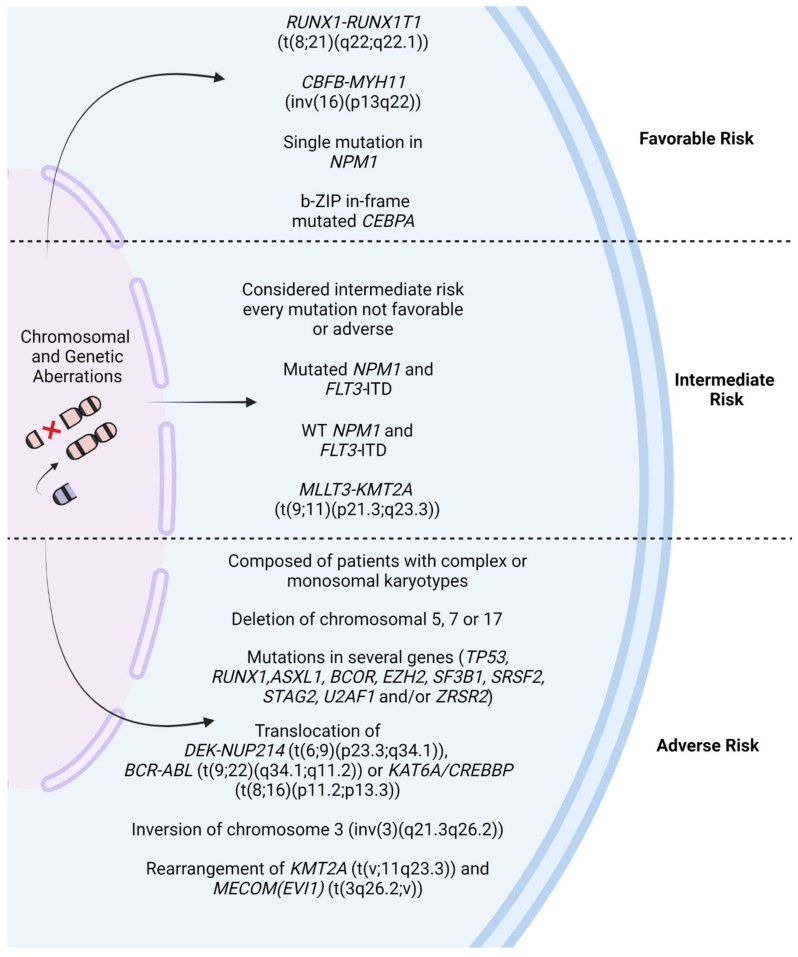
**Cytogenetic and chromosomal abnormalities associated with prognostic risk groups in acute myeloid leukemia (AML).** RUNX1: RUNX family transcription factor 1; RUNX1T1: RUNX1 partner transcriptional co-repressor 1; CBFB: core-binding factor subunit beta; MYH11: myosin heavy chain 11; CEBPA: CCAAT enhancer binding protein alpha; NPM1: nucleophosmin 1; bZIP: basic leucine zipper domain; FLT3-ITD: fms-related receptor tyrosine kinase 3—internal tandem duplication; WT: wild-type; KMT2A: lysine methyltransferase 2A; MLLT3: MLLT3 super elongation complex subunit; TP53: tumor protein p53; ASXL1: ASXL transcriptional regulator 1; BCOR: BCL6 corepressor; EZH2: enhancer of zeste 2 polycomb repressive complex 2 subunit; SF3B1: splicing factor 3b subunit 1; SRSF2: serine and arginine rich splicing factor 2; STAG2: stromal antigen 2; U2AF1: U2 small nuclear RNA auxiliary factor 1; ZRSR2: zinc finger CCCH-type, RNA-binding motif and serine/arginine rich 2; DEK: DEK proto-oncogene; NUP214: nucleoporin 214; BCR: BCR activator of RhoGEF and GTPase; ABL: ABL proto-oncogene 1; KAT6A: lysine acetyltransferase 6A; CREBBP: CREB binding protein; KMT2A: histone-lysine N-methyltransferase 2A; MECOM (EVI1): ecotropic virus integration site 1 protein homolog. Created with Biorender.com.

The European Leukemia Net (ELN) and World Health Organization (WHO) guidelines are probably the most widely used sources for assessing the risk of resistance and classifying patients into favorable, intermediate, and adverse groups on the basis of leukemia cell cytogenetics and mutations (Figure 1) [14,15]. The main cytogenetic–molecular entities in AML are translocations 15;17 (t(15;17)(q22,q21)), 8;21 (t(8;21)(q22;q22.1)), and 9;22 (t(9;22)(q34.1;q11.2)); inversions 16 (inv(16)(p13q22) or t(16;16)(p13;q22)) and 3 (inv(3)(q21.3q26.2) or t(3;3)(q21.3;q26.2)); mutations in *TP53*, *NPM1*, *FLT3*, and *IDH*; complex karyotypes; and others.

### 2.1. Favorable Risk Genetic Alterations

Core-binding factor (CBF) is a transcription factor that plays critical roles in hematopoietic stem cell (HSC) maintenance and differentiation. CBF has the ability to regulate the expression of genes associated with cellular proliferation and death. In general, this factor is characterized by the presence of either t(8;21) (q22;q22) or inv(16)(p13q22), which leads to the formation of fusion genes that are associated with a favorable response to treatment (*RUNX1-RUNX1T1* and *CBFB-MYH11*, respectively) [16,17,18].

According to the ELN guidelines, patients who have mutations in CBF, such as translocation 8;21 (t(8;21)(q22;q22.1)) and inversion 16 (inv(16)(p13q22) or t(16;16)(p13;q22)), are classified as having favorable risk, along with patients with a single mutation in *NPM1* or bZIP in-frame mutated *CEBPA* [14].

The translocation t(8;21)(q22;q22.1) results in a gene fusion between the RUNX1 and RUNX1T1 genes, deriving the *RUNX1-RUNXT1* hybrid gene. This cytogenetic aberration is especially identified in patients with M2 AML according to the French–American–British classification (FAB) [19]. Due to high rates of complete remission after conventional treatments, the presence of this translocation came to be considered a good prognostic factor and an indicator of favorable outcomes for patients [16,20,21,22].

The inversion inv(16)(p13q22) and the translocation t(16;16)(p13q22) result in the formation of the chimeric gene *CBFB-MYH11*, which encodes the fusion protein CBFb-SMMHC. The inv(16) mutation is reported in almost all patients with the M4Eo (M4 with eosinophilia) AML subtype according to FAB, which constitutes about 5–8% of all patients with AML. Just like *RUNX1-RUNXT1*, *CBFB-MYH11* is also considered a cytogenetic alteration that confers a favorable prognosis in afflicted patients [23,24,25,26].

Mutations in the *CEBPA* gene are present in 10–15% of AML patients. The *CEBPA* gene is located on chromosome 19 and encodes CCAAT enhancer binding protein alpha (CEBPa), which is a crucial transcription factor for stem and progenitor cell function and the regulation of myeloid cell differentiation. CEBPa mutations in the basic leucine zipper domain (bZIP) have been associated with a favorable prognosis; they are an indicator of a higher chance of achieving complete remission (CR), better overall survival (OS), and a lower risk of relapse [27,28,29,30,31,32,33]. 

The nucleophosmin (*NPM1*) gene is located on chromosome 5 and encodes a protein that is involved in critical cell function by playing key roles in ribosome biogenesis, centrosome duplication, genomic stability, cell cycle progression, and apoptosis. *NPM1* is the most commonly mutated gene in adult AML, occurring in approximately 30% of all cases, and it is considered to be a prognostic factor of favorable risk [15,34,35,36,37,38,39,40].

### 2.2. Intermediate Risk Genetic Alterations

The ELN defines intermediate-risk patients as those with cytogenetic abnormalities not qualified as favorable or adverse, with mutated *NPM1* co-occurring with a low level of *FLT3-ITD* or wild-type *NPM1* with the co-occurrence of *FLT3-ITD* and the translocation (t(9;11)(p21.3;q23.3)) [41].

The detection of the *FLT3-ITD* mutation is documented in about 40% of *NPM1*-mutated AML cases. The deleterious prognostic effects of *FLT3-ITD* have previously been found to be most clinically relevant when co-occurring with *NPM1* mutations, which are characterized by a higher relapse rate and poorer OS [35,37,42,43].

In AML, translocation 9;11 (t(9;11)(p22;q23)) results in the *KMT2A-MLLT3* fusion protein. This translocation is the most common one involving *KMT2A*, and it can be found in some types of AML, such as de novo and therapy-related AML (t-AML). Usually, the presence of *KMT2A-MLLT3* fusion is correlated with a poorer prognosis and more complex cytogenetics [43,44,45].

### 2.3. Adverse Risk Genetic Alterations

According to the ELN, the high-risk group is composed of patients with complex or monosomal karyotypes; inversion of chromosome 3 (inv(3)(q21.3q26.2)); rearrangement of *KMT2A* (t(v;11q23.3)) or *MECOM(EVI1)* (t(3q26.2;v)); translocations 6;9 (1(6;9)(p23.3;q34.1)), 9;22 (t(9;22)(q34.1;q11.2)), and 8;16 (t(8;16)(p11.2;p13.3)); deletions of chromosome 5, 7, or 17; or mutations in several genes, such as T*P53*, *RUNX1*, *ASXL1*, *BCOR*, *EZH2*, *SF3B1*, *SRSF2*, *STAG2*, *U2AF1,* and/or *ZRSR2* [14,41,43].

The translocation t(6;9)(p23;q34) generates the fusion gene *DEK-NUP214*. It is a relatively rare mutation in AML and is associated with a poorer prognosis. Most cases reported in the literature are commonly found in AML M2 and M4 according to FAB classification, although this alteration can also be identified in AML M1 in a few cases [46,47,48].

The t(9;22) chromosome translocation originating from the *BCR-ABL1* fusion oncoprotein is detected most commonly in chronic myeloid leukemia (CML), occurring in approximately 95% of the cases and representing the hallmark of this disease. However, t(9;22) can also be found in 10 to 20% of adults and 2 to 5% of children with acute lymphoblastic leukemia (ALL), as well as in rare cases (1% approximately) of AML. The prognosis of *BCR-ABL*-mutated AML seems to depend on the cytogenetic and/or molecular background rather than *BCR-ABL* itself. Cases of AML with this mutation can be often challenging to distinguish from the presentation of CML in a blast crisis [49,50,51].

The *TP53* protein has an impact on various cellular functions, especially DNA repair and cellular senescence, and the inactivation of *TP53* by either mutation or deletion increases the proliferation of cancer cells. In AML, mutated *TP53* is reported in patients with older age, lower blast counts, adverse risk karyotypes, and exposure to antecedent chemotherapy. Overall, this genetic aberration may occur across nearly all FAB subtypes. The *TP53* mutation associated with AML represents a distinct subgroup associated with a poorer prognosis since this mutation predicts inferior OS and resistance to cytotoxic chemotherapies [52,53,54].

Notably, all these genetic aberrations have an impact on the patient’s diagnosis, treatment, and prognosis. Thus, the development of novel targeted therapies that may help deal with these cytogenetic alterations in a specific and individualized way is essential.

## 3. Immunophenotyping in Acute Myeloid Leukemia Cells

The antigens that summarize the immunophenotype of a cell are the indicators of its identity, making it possible to characterize its degree of maturation and lineage; the main surface antigens of myeloid lines are CD11b, CD13, CD14, CD15, and CD33. From the phenotypic alterations that occur in a neoplastic clone, the surface antigens also undergo changes that can be used to identify and characterize these cells. Due to the variability of immunophenotypic changes present in leukemic cells, they can be categorized into groups: expression of crossline antigens, asynchronous expression of maturational markers, expression of decreased or absent antigens, and overexpression of antigens [21,55,56].

For AML, diagnostic tools include cytomorphology, cytochemistry, immunophenotyping, cytogenetics, and molecular genetics techniques, all of which complement each other to classify the disease in view of its intrinsic heterogeneity. However, among these techniques, immunophenotyping is of great importance, as it is crucial for the detection, characterization, and quantification of cells and can provide a useful line of markers for differential diagnosis and classification. Noting that leukemic cells have immunophenotypic aberrations, this technique allows the identification of acute leukemias of mixed lineage while also being able to suggest possible genetic changes [57,58,59]. 

Regarding the identification of antigens for the diagnosis of AML, they are classified as precursors (CD34, CD117, HLA-DR), myeloid markers (cMPO, CD33, CD13), myeloid maturation markers (CD11b, CD15, CD64, CD4, CD38, CD11c), monocytic markers (CD14, CD36, CD64), megakaryocytic markers (CD41, CD36, CD61), and erythrocyte markers (CD235, CD71, CD36) [14,57,60,61].

Immunophenotyping also plays a fundamental role in the evaluation of treatment response and the detection of minimal/measurable residual disease (MRD), for which the identification of antigens defined at the time of diagnosis is sought and their presence is evaluated during and/or after treatment [3,62].

MRD can be performed at two moments: right after the beginning of the induction of treatment (to evaluate the kinetics of the response to the disease) and after the end of treatment (to evaluate the possibility of recurrence). The means of detection of MRD have evolved with advances in therapies, and today, the approaches used for its evaluation are multiparametric flow cytometry (MFC) and molecular approaches, such as real-time quantitative PCR (qPCR), digital PCR (dPCR), and next-generation sequencing (NGS) [63,64,65].

Advances in diagnostic techniques have led to improvements in not only the prognosis and monitoring of the disease but also the classification of AML with the precise identification of mutations. However, the application of MFC and qRT-PCR/NGS in combination still needs to be better investigated in order for it to become a feasible approach in AML [57,64].

## 4. Immunophenotyping x Genetics in AML

Identifying correlations between immunophenotypic markers and genetic changes may provide more a precise diagnosis and better targeted treatment strategies for AML patients. In this context, Table 1 comprises a series of clinical trials with published results that, in the past 10 years, described genetic alterations and/or clinical features and immunophenotypic characteristics at diagnosis. These studies were found in the PubMed database from research using the descriptors “acute myeloid leukemia”, “immunophenotyping”, “genetic alterations”, and “prognosis”.

Currently, the wide application of immunophenotyping and genetic characterization has become critical for clinicopathological evaluations, diagnostic refinement, risk group classification, prognosis prediction, and promotion of targeted therapy in AML patients [66,67,68].

**Table 1:** Studies from the last 10 years indicating relationships between immunophenotyping and genetic changes in AML patients.

Number of Patients	Mean Age	Immunophenotypic Characteristics	Genetic Alterations	References
41 (71% males and 29% females)	66	CD45, CD34, CD117, CD36, HLA-DR, CD13, CD33, CD7, CD71	Deletions involving chromosome 5/5q; deletions involving chromosome 7/7q; deletion of the TP53 locus (17p13)	[69]
90 patients	Aged > 60 years	CD123, CD34, CD7, CD117, CD64, CD38, CD11b, CD33, CD56	TET2, DNMT3A, IDH2, NPM1, FLT3-ITD, CEBPA, ASXL1, IDH1, SRSF2, BCOR, TP53, NRAS, RUNX1, U2AF1, BCORL1, WT1, and FLT3-TKD.	[70]
67 (26 males and 41 females)	64 (19–84)	CD34, CD117, HLA-DR	NPM1 p. W288fs DNMT3A, FLT3, TET2, IDH2, PTPN11, IDH1, NRAS, SRSF2, RAD21, WT1, ASXL1, and NF1	[71]
1040 (51.92% males and 48.08% females)	10.3	CD123	t(8;21), inv(16), CEBPA, NPM1, FLT3-ITD; monosomy 7, monosomy 5/del(5q), t(6;9) with DEK-NUP214 fusion, KMT2A rearrangements	[72]
37 Males: 20 (54%), Females: 17 (46%)	54 (24–70)	CD8+CD45RA− CD27+/int CD28+ PD1+ TCF1+	NPM1, DNMT3A, ASXL1, IDH2, TP53, CEBPA, NRAS, WT1	[73]
84 (51 females and 33 males)	65.5 (22–89)	CD34-, HLA-DR-, CD117+, CD11b-, CD56+, CD13	FLT3-ITD, FLT3-TKD, DNMT3A, NPM1, TET2, IDH1, IDH2	[74]
769 patients	NR	CD56, CD34, CD7, CD11b, CD117, CD13, SSC, CD33, CD45, CD38, FSC, HLA-DR	FAB, inv(16), t(8;21), cKit, 11q23, FLIT3, NPM1, CEBPA, HT1, GLIS2	[75]
217 patients	NR	CD34+ CD38− CD90− CD45RA+	RUNX1 DNMT3A IDH2 TET2	[76]
174 (153 adults and 21 children	Adults aged 20–85 years and children aged < 20 years	CD34, CD7, HLA-DR	NPM1, FLT3-ITD, FLT3-D835, DNMT3A, NPM1	[77]
13 (11 males and 2 females)	68	CD13+, CD34+, HLA-DR+, CD33	addX, del7, del5/5q, and add19	[78]

AML: acute myeloid leukemia; CD: cluster of differentiation; HLA-DR: human leukocyte antigens; NR: not reported; ITD: internal tandem domain; TKD: tyrosine kinase domain; ADD: addition; DEL: deletion; NT/T: near-tetraploidy/tetraploidy; APL: acute promyelocytic leukemia.

All 10 articles described in Table 1 reported the main genetic alterations and the immunophenotyping results of AML patients enrolled in each study. Of all the reported cytogenetic abnormalities, the most frequent were mutations involving NPM1 (7), FLT3 (7), and DNMT3A (6). Regarding immunophenotyping results, the most frequent immunophenotyping markers were CD34 (7), HLA-DR (5), CD45 (4), and CD13 (4) [69,70,71,72,73,74,75,76,77,78].

The *NPM1* mutation was one of the most frequent genetic aberrations detected in the populations of the different studies (7/10), and it is indeed the most frequent in AML cases, usually representing a favorable prognosis when presented as a single mutation [14,70,71,72,73,74,75,77]. However, as previously stated, these mutations can be commonly associated with mutations in other genes, such as *DNMT3A*, *FLT3*, *TET2*, *IDH1,* and *IDH2*, all of which directly influence AML leukemogenesis progression [71,76]. It was possible to observe a significant relationship between the mutation of the *NPM1* gene and a lower expression of CD34+ or CD34 expression, indicating a favorable prognosis in patients with this profile, as observed by Dohner et al. in 2005 [79]. 

In addition, CD34+ expression in more than 10% of blast cells and/or leukemic stem cells (LSCs) is reported in approximately 75% of cases of AML. However, in other cases in which this expression does not occur, it is possible to observe *NPM1* mutations [61,70,71,77,79]. Some studies have associated NPM1-mutated AML with CD117+ blasts with increased expression of CD33 and/or CD123 and decreased expression or absence of CD13, HLA-DR, CD15, and CD64, along with CD34 [80,81].

Mutated *FLT3* was reported in 7 of the 10 studies in Table 1 [70,71,72,73,74,75,77]. Although mutations may manifest as either internal tandem duplication of the juxta membrane domain (FLT3-ITD) or point mutations in the tyrosine kinase domain (FLT3^TKD^), FLT3-ITD is the manifestation more highly associated with a worse prognosis in AML patients, predicting shorter remission and decreased OS [82,83]. Some studies have identified that the phenotypes CD34, CD123, CD25, and CD99 predict both FLT3-ITD and FLT3-ITD^mut^. This correlation could lead to the utilization of targeted therapies with specific inhibitors [84,85]. Furthermore, higher rates of FLT3-ITD mutations were associated with a higher expression of the CD33 phenotype in the study of Feldman et al. [86], leading to a shorter duration of remission [87,88,89].

**Figure 2:**
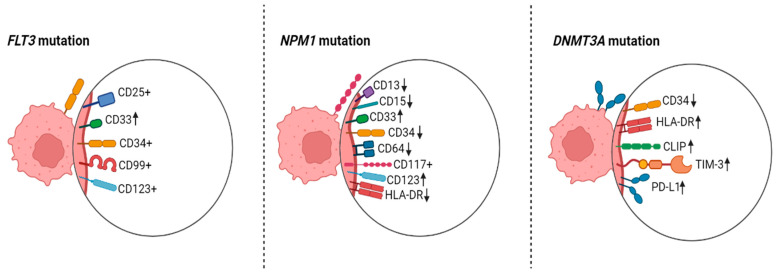
Commonly reported genetic mutations and associated immunophenotypic characteristics in acute myeloid leukemia (AML) neoplastic clones. CD: cluster of differentiation; DNMT3A: DNA methyltransferase 3 alpha; FLT3: fms-related receptor tyrosine kinase; HLA-DR: major histocompatibility complex, class II, DR; NPM1: nucleophosmin 1; PD-L1: programmed cell death 1 ligand 1; TIM-3: T-cell immunoglobulin mucin family member 3. Created with Biorender.

Alterations in *DNMT3A* were present in patients in five different studies described in Table 1, and they are also common mutations in early leukemic stages that confer proliferative advantages to pre-leukemic cells [56]. *DNMT3A* activity on gene methylation directly influences AML development, as either hypermethylation of tumor suppressors or hypomethylation of oncogenes might act as drivers of leukemogenesis onset [90]. However, although many mutations in *DNMT3A* are cataloged, few have been thoroughly investigated, with point mutations in the catalytic domain being the major players associated with AML drivers [91]. Other studies have shown that patients with *DNMT3A* mutation present higher levels of HLA- DR, CLIP, PD-L1, and TIM-3 and lower levels of CD34 (Figure 2) [89,92,93].

The majority of studies in Table 1 (7/10) reported the presence of CD34+ immunophenotypic markers in most patients. Its presence is associated with immature cell populations, worse prognosis due to increased cell proliferation, blockage in differentiation, cell traffic and adhesion pathways, and increased resistance to induced apoptosis when compared with CD34- cells [69,70,71,75,76,77,78]. In a study by Loghavi et al. [71], for example, it was demonstrated that CD34+ expression combined with a CD117 increase and a CD38 and/or HLA-DR decrease may characterize complications in AML. Both the HLA-DR and CD117 markers are found in myeloid precursor cells and activate metabolization by inducing cycle progression; CD117 is a primitive myeloid marker associated with a worse prognosis. In turn, the negative expression of HLA-DR is associated with a favorable evolution and complete remission unless it is associated with CD117+, which is then correlated with unfavorable cytogenetics [94,95].

CD123 is associated with high-risk disease in AML, with greater expression in patients with KMT2A rearrangements and FLT3-ITD mutations, as observed by Lamble et al. [72]. As an interleukin 3 (IL-3) receptor, the CD123 plays an important role in the production and function of healthy hematopoietic cells, but its overexpression in leukemic patients results in an adverse prognosis, with leukocytosis due to increased susceptibility to IL-3 and higher chemoresistance rates due to the high replication rate. In addition, mutations in DNMT3A and WT1 can also be strongly associated with CD123 [70,96,97,98,99,100].

Some cytogenetic alterations already have a well-established immunophenotype in the literature, such as AML patients with PML-RARA, RUNX1-RUNX1T1, and CBFB-MYH11, as shown in Table 2, where red and green are consistent with the increase and decrease in the markers’ expression, respectively. Studies on PML-RARA AML have identified the positive expression of CD33, CD13, and CD19 antigens; the absence of HLA-DR expression; and low-frequency CD7, CD11b, and CD14 expression. Moreover, aberrant surface antigens, including CD2 and CD34, have also been described in some variations of APL [101,102,103]. The common blast immunophenotypes of RUNX1-RUNX1T1 AML were CD34, CD117, CD13, CD33, CD19, CD56, CD38, HLA-DR, and MPO [104,105,106]. Regarding the CBFB-MYH11 fusion gene, studies have reported a high frequency of CD33, CD34, CD117, HLA-DR, CD15, CD64, and CD14 expression [107,108]. 

## 5. Conclusions

Studies have pointed out that it is possible to have a correlation between genetic alterations and surface antigens in patients with AML; however, further studies are needed to establish a complete correlation between alterations and markers. Such information may provide a faster diagnosis for AML patients due to the easy access to immunophenotyping tools, which could help steer investigators in clinical practice toward a preset cohort of genetic alterations while also highlighting which targeted therapeutic strategies may potentially be more effective for patient treatment.

## Figures and Tables

**Table 2:** Correlation between mutations and immunophenotypic profiles.

	*RUNX1-RUNX1T1*	*PML-RARA*	*CBFB-MYH11*	*FLT3*	*NPM1*	*DNMT3A*
CD34						
CD117						
CD19						
CD13						
CD33						
HLA-DR						
MPO						
CD7						
CD123						
CD64						
CD38						
CD11b						
CD56						
CD14						
CD2						
CD15						
CD25						
CD99						

CD: cluster of differentiation; HLA-DR: human leukocyte antigens; MPO: myeloperoxidase; RUNX1: RUNX family transcription factor 1; RUNX1T1: RUNX1 partner transcriptional co-repressor 1; CBFB: core-binding factor subunit beta; MYH11: myosin heavy chain 11; NPM1: nucleophosmin 1; FLT3-ITD: fms-related receptor tyrosine kinase 3—internal tandem duplication; DNMT3A: DNA methyltransferase 3 alpha; Red: increased marker expression; Green: decreased marker expression.
